# Pro-Inflammatory and Neurotrophic Factor Responses of Cells Derived from Degenerative Human Intervertebral Discs to the Opportunistic Pathogen *Cutibacterium acnes*

**DOI:** 10.3390/ijms22052347

**Published:** 2021-02-26

**Authors:** Manu N. Capoor, Anna Konieczna, Andrew McDowell, Filip Ruzicka, Martin Smrcka, Radim Jancalek, Karel Maca, Michael Lujc, Fahad S. Ahmed, Christof Birkenmaier, Stefan Dudli, Ondrej Slaby

**Affiliations:** 1 Laboratory of Bacterial Pathogenesis and Immunology, Rockefeller University, 1230 York Avenue, New York, NY 10065, USA; 2 Central European Institute of Technology (CEITEC), Masaryk University, 625 00 Brno, Czech Republic; anusia@email.cz (A.K.); fsa526@gmail.com (F.S.A.); 3 Nutrition Innovation Centre for Food and Health (NICHE), School of Biomedical Sciences, Ulster University, Coleraine BT52 1SA, UK; a.mcdowell@ulster.ac.uk; 4 Department of Microbiology, Faculty of Medicine, St. Anne’s University Hospital, Masaryk University, 656 91 Brno, Czech Republic; filip.ruzicka@fnusa.cz; 5 Department of Neurosurgery, University Hospital Brno, Masaryk University, 625 00 Brno, Czech Republic; smrcka.martin@fnbrno.cz (M.S.); maca.karel@fnbrno.cz (K.M.); 6 Department of Neurosurgery, St. Anne’s University Hospital, Masaryk University, 656 91 Brno, Czech Republic; radim.jancalek@fnusa.cz; 7 Department of Orthopaedic Surgery, University Hospital Brno, Masaryk University, 625 00 Brno, Czech Republic; michael.lujc@seznam.cz; 8 Department of Orthopaedic Surgery, Physical Medicine and Rehabilitation, University of Munich, 80331 Munich, Germany; christof.birkenmaier@med.uni-muenchen.de; 9 Centre of Experimental Rheumatology, Department of Rheumatology, University Hospital, University of Zurich, 8091 Zurich, Switzerland; stefan.dudli@usz.ch; 10 Department of Physical Medicine and Rheumatology, Balgrist University Hospital, University of Zurich, 8091 Zurich, Switzerland; 11 Department of Biology, Faculty of Medicine, Masaryk University, 601 77 Brno, Czech Republic

**Keywords:** *Cutibacterium acnes*, disc cells, co-culture, inflammation, neurotrophic factors, gene expression, intracellular

## Abstract

Previously, we proposed the hypothesis that similarities in the inflammatory response observed in acne vulgaris and degenerative disc disease (DDD), especially the central role of interleukin (IL)-1β, may be further evidence of the role of the anaerobic bacterium *Cutibacterium* (previously *Propionibacterium*) *acnes* in the underlying aetiology of disc degeneration. To investigate this, we examined the upregulation of IL-1β, and other known IL-1β-induced inflammatory markers and neurotrophic factors, from nucleus-pulposus-derived disc cells infected in vitro with *C. acnes* for up to 48 h. Upon infection, significant upregulation of IL-1β, alongside IL-6, IL-8, chemokine (C-C motif) ligand 3 (CCL3), chemokine (C-C motif) ligand 4 (CCL4), nerve growth factor (NGF) and brain-derived neurotrophic factor (BDNF), was observed with cells isolated from the degenerative discs of eight patients versus non-infected controls. Expression levels did, however, depend on gene target, multiplicity and period of infection and, notably, donor response. Pre-treatment of cells with clindamycin prior to infection significantly reduced the production of pro-inflammatory mediators. This study confirms that *C. acnes* can stimulate the expression of IL-1β and other host molecules previously associated with pathological changes in disc tissue, including neo-innervation. While still controversial, the role of *C. acnes* in DDD remains biologically credible, and its ability to cause disease likely reflects a combination of factors, particularly individualised response to infection.

## 1. Introduction

Degenerative disc disease (DDD) is amongst the most common causes of chronic lower back pain (CLBP), placing a tremendous burden on healthcare systems [1]; CLBP affects over 400 million individuals globally [2]. Factors related to disc degeneration range from biomechanical and exogenous factors, such as nutrition and smoking, to genetic predisposition and changes in disc architecture due to ageing [2,3,4,5]. Treatment strategies are difficult to devise and often produce less than satisfactory results, underscoring the need for better diagnostic tools and a more complete understanding of the mechanisms underlying DDD. In particular, the identification of new and potentially reversible factors which affect the degenerative process are highly desirable, potentially leading to new tangible treatment strategies.

Over the last 20 years, many studies, although not all, have linked the anaerobic skin bacterium *Cutibacterium* (previously *Propionibacterium*) *acnes* with degenerated disc tissue, thus suggesting that low-grade *C. acnes* infection of the intervertebral disc (IVD) may be an important factor in the development of DDD [6,7,8,9,10,11]. While the association of *C. acnes* with DDD remains controversial, the bacterium has been shown to exist with a deeply embedded biofilm-like morphology in disc tissue, demonstrating that dismissal of the organism as a skin contaminant in all cases is too simplistic [8,10]. Furthermore, evidence of a pathogenic involvement in DDD has come from experimental animal studies, some of which have also demonstrated the capacity of the bacterium to simulate Modic-like changes upon infection [12,13,14,15]. The demonstration that symptoms of CLBP in patients with Modic type I (bone oedema) can be successfully alleviated with a ~3-month course of amoxicillin-clavulanate also provides additional circumstantial support for a potential bacterial-driven aetiology, although this efficacy of antibiotics has been disputed [16,17]. While there is a growing body of evidence supporting the involvement of *C. acnes* in DDD, a definitive causal role is challenging to prove since the bacterium comprises part of the normal human microbiota on the skin and other sites during health, thus making fulfilment of Koch’s original stated postulates impossible [18]. Acceptance of *C. acnes* as a contributing factor to DDD in some patients will, therefore, require embracing modifications of these postulates [18] and the collection of further evidence drawn from epidemiological and clinical-trial investigations, as well as additional animal and cell-culture-based infection studies.

DDD is associated with cellular, extracellular and inflammatory changes in the IVD [19,20]; the latter is believed to be critical in its aetiology. Interleukin (IL)-1β, which is produced via nod-like receptor family pyrin domain containing 3 (NLRP3)-inflammasome activation of pro-IL-1β, helps drive T helper (Th) lymphocyte 1 (Th1) and Th17-induced immune responses and is highly expressed in degenerative discs, where it appears to act as a master regulator of catabolic processes [20,21,22]. In particular, it can induce tissue destruction via matrix metalloprotease and aggrecanase production from IVD cells, as well as stimulation of other cytokines and chemokines [21,23,24,25]. In the latter case, these include chemokine (C-C motif) ligand 3 (CCL3) and chemokine (C-C motif) ligand 4 (CCL4), which are involved in the recruitment and activation of infiltrating immune cells [21,26]. It can also promote angiogenesis, apoptosis and acceleration of cellular senescence [27] and stimulate the expression of nerve growth factor (NGF) and brain-derived neurotrophic factor (BDNF) in disc cells, leading to acceleration of DDD and neo-innervation [28,29,30,31]; increases in nucleus pulposus (NP) cell expression of BDNF have, however, not always been observed upon IL-1β treatment [32]. The inflammatory processes in DDD provide some interesting parallels with those observed in the common skin condition acne vulgaris, which has had a long association with *C. acnes* in its pathophysiology [33]. In particular, IL-1β is also highly abundant in its active form within inflammatory lesions where monocyte-macrophage NLRP3-inflammasome activation is triggered by *C. acnes* [34]. Th1 and Th17 response pathways are also stimulated, and we see an infiltration of CD68+, CD4+ and CD8+ inflammatory cells, similar to that observed in herniated discs [33,35,36].

We previously described the intriguing hypothesis that many of the shared inflammatory features between DDD and acne, especially the central role of IL-1β, could be explained, at least in part, by a common role for *C. acnes* in the pathology of both conditions and that *C. acnes* as a driver of DDD is biologically plausible [37]. To build a case that the bacterium may be a key stimulant of IL-1β production in the diseased discs of some patients, we described how a series of host–pathogen experiments could be performed, including investigation of IL-1β expression patterns by (NP)-derived disc cells upon infection with *C. acnes* [37].

The aim of this current study was, therefore, to investigate in vitro whether *C. acnes* can act as a stimulator of IL-1β from cells prepared from degenerative human discs, as well as other IL-1β-induced cytokines, chemokines and neurotrophic factors which could contribute to discogenic pain. 

## 2. Results

### 2.1. Detection of C. acnes in Disc Tissue

The donor tissues from all eight patients showed no evidence of *C. acnes* infection based on agar plate culture and, therefore, were deemed *C. acnes*-naive cells.

### 2.2. C. acnes Appears Intracellularly upon Infection 

**Figure 1:**
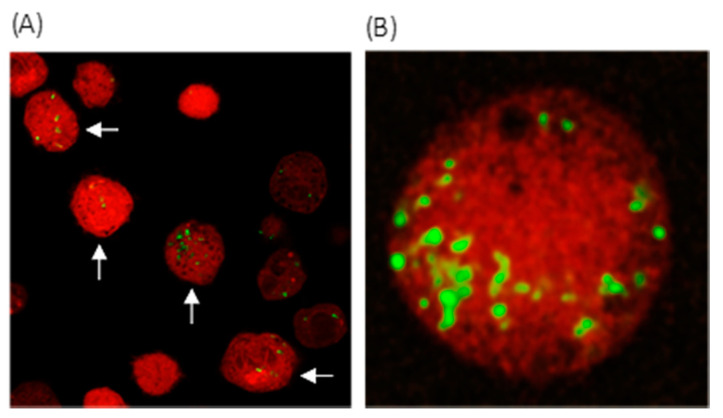
Confocal microscopy image of *C. acnes* infected KB7007NP disc cells (arrows) [multiplicity of infection (MOI) 1:1000; 48 h, original multiplication x40] (**A**). Fluorescently labelled *C. acnes* (green) were visible as intracellular clusters, and individual bacteria within viable Calcein Red-Orange-stained disc cells derived from NP tissue (**B**).

Fluorescently labelled *C. acnes* was observed as intracellular clusters and individual bacteria [at both multiplicities of infection (MOIs)] within viable disc cells at the 48 h incubation period (Figure 1A,B); disc cell viability was demonstrated by the uptake of Calcein Red-Orange dye, with cells displaying a characteristic rounded morphology. 

### 2.3. Cytokine and Chemokine Gene Expression 

**Figure 2:**
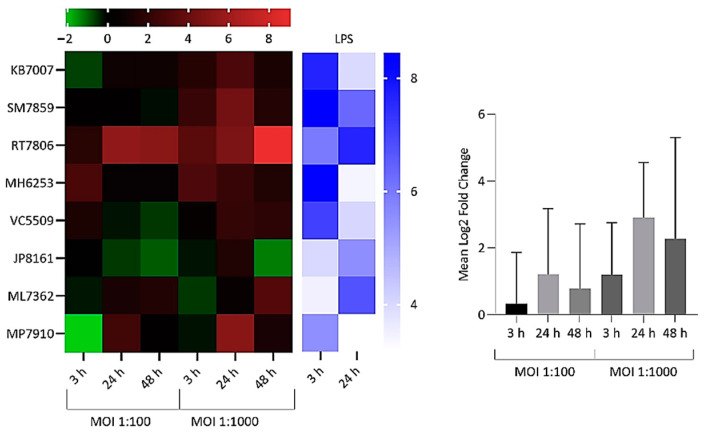
Heatmap and bar chart of log_2_ fold gene expression changes for interleukin (IL)-1β in NP-derived disc cells infected with *C. acnes*. Heatmap values are individual expression changes (3 × biological replicates) from eight donor samples, while the bar chart represents average donor changes. Results with lipopolysaccharide (LPS)-positive controls are also shown. The top bar represents the log_2_ fold-change scale.

**Figure 3:**
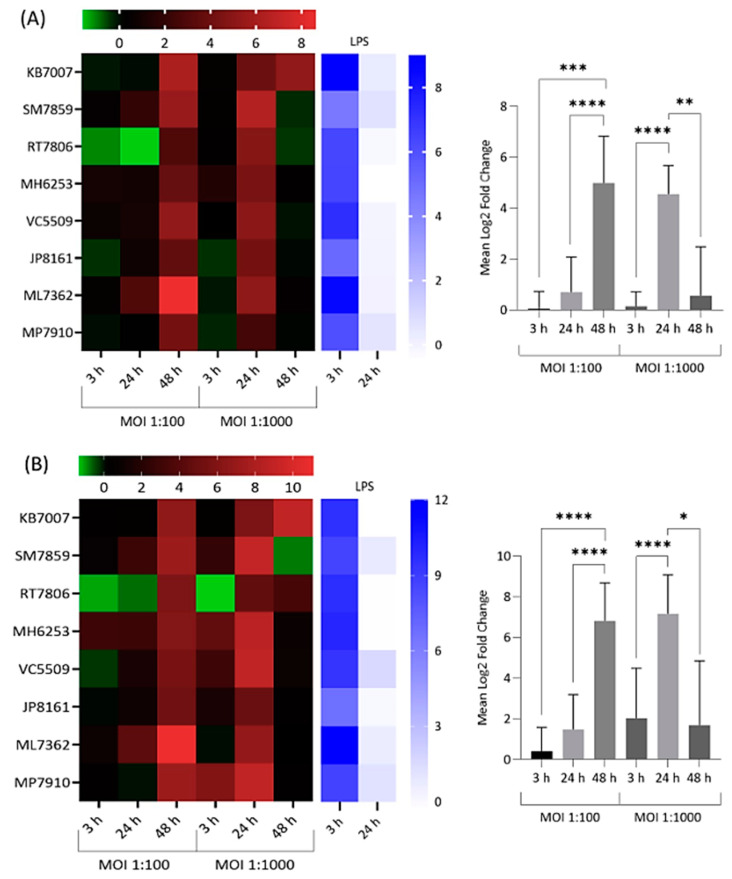
Heatmap and bar chart of log_2_ fold gene expression changes for IL-6 (**A**) and IL-8 (**B**) in NP-derived disc cells infected with *C. acnes*. Heatmap values are individual changes (3 × biological replicates) from eight donor samples, while the bar charts represent average donor changes. Results with LPS-positive controls are also shown. Top bars represent the log_2_ fold-change scale * *p* < 0.05; ** *p* < 0.01; *** *p* < 0.001; **** *p* < 0.0001.

**Figure 4:**
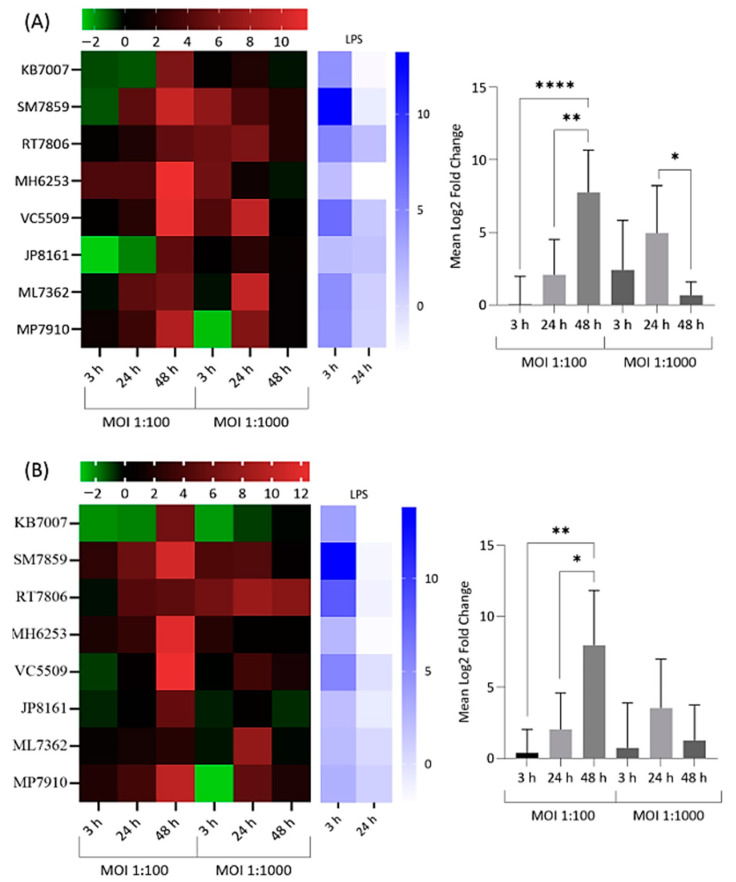
Heatmap and bar chart of log_2_ fold expression changes for chemokine (C-C motif) ligand 3 (CCL3) (**A**) and chemokine (C-C motif) ligand 4 (CCL4) (**B**) in NP-derived disc cells infected with *C. acnes*. Heatmap values are individual changes (3 × biological replicates) from eight donor samples, while the bar chart represents average donor changes. Results with LPS positive controls are also shown. Top bars represent the log_2_ fold-change scale * *p* < 0.05; ** *p* < 0.01; **** *p* < 0.0001.

Significant upregulation in the expression of all targeted cytokines and chemokines by infected and lipopolysaccharide (LPS)-treated NP-derived disc cells was observed at various incubation time points versus mock-infected negative controls (Figure 2, Figure 3 and Figure 4). The response to infection was, however, variable with individual levels of gene dysregulation dependent on the patient, the target analysed, the MOI and the sampling time point (Figure 2, Figure 3 and Figure 4). 

For IL-1β, significant increases in gene expression were observed amongst infected cells from donors versus controls, with stronger responses seen at MOI = 1:1000. Levels of overall expression broadly increased with time, but this did not reach statistical significance (Figure 2). Donor cells from RT7806 were the most sensitive or consistent responders in terms of IL-1β expression, producing the highest fold change of approximately 500 at 48 h with MOI = 1:1000. In contrast, JP8161 cells were consistently weak responders. 

With IL-6 and IL-8 inflammatory markers, a large peak in acute expression was observed across all donor cells at 48 h for MOI = 1:100 and 24 h for MOI = 1:1000 (Figure 3). 

The average levels of expression at these IL-6 and IL-8 peaks were significantly different from pre- and/or proceeding sampling times and also greater than those observed with IL-1β, which gave more moderate responses overall (Figure 2).

Significant upregulation in the gene expression of CCL3 and CCL4 was also detected amongst infected donor cells versus controls, with MOI = 1:100 stimulating the highest values overall (Figure 4). In the latter case, average expression values spiking at 48 h were significantly higher than preceding sampling times (Figure 4). Donor cells MH6253, VC5509 and SM7859 gave the highest expression for both chemokines at this time point and MOI. RT7806 cells, although not the highest CCL4 expressers on infection, did demonstrate a sustained response over time versus other donors (Figure 4B).

### 2.4. Correlation between IL-1β Gene Expression and Other Cytokines and Chemokines 

**Figure 5:**
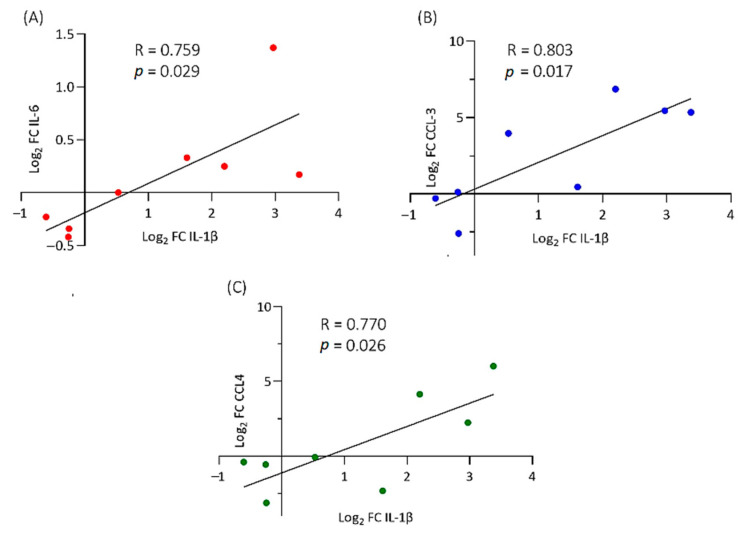
Statistically significant correlation between IL-1β and IL-6 (**A**), CCL3 (**B**) and CCL4 (**C**) gene expression [Fold change (FC)] after 3 h infection (MOI = 1:1000). Data represent eight donor cell samples.

Given our interest in IL-1β and its potential to act as a master regulator and key autocrine and paracrine signal for the production of other cytokines and chemokines in disc disease, we examined whether any correlation could be established between levels of IL-1β gene expression and IL-6, IL-8, CCL3 and CCL4 across all eight donor cell samples infected with *C. acnes*; these inflammatory mediators are known to be stimulated by IL-1β. With cells infected at MOI = 1:100, no significant correlations between different gene expression levels were observed at any of the time points. However, at MOI = 1:1000, moderate correlations were detected at 3 h between IL-1β and IL6,CCL3 and CCL4, but these associations were found to disappear from 24 h onwards (Figure 5). 

### 2.5. Protein Expression

**Figure 6:**
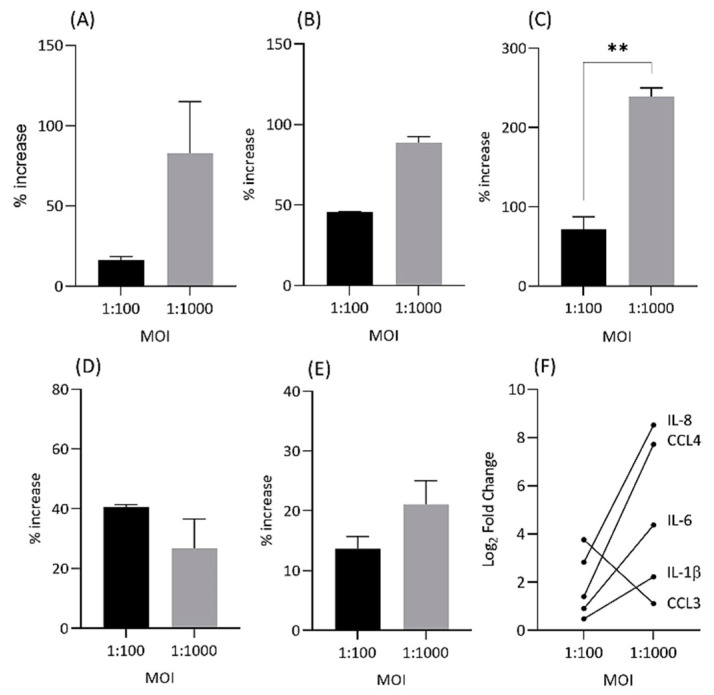
Percentage increase in protein expression (ng/mL) for IL-1β (**A**), IL-6 (**B**), IL-8 (**C**), CCL3 (**D**) and CCL4 (**E**) at 24 h and MOIs = 1:100 and 1:1000 for infected vs. control NP cells. Changes in gene expression levels between MOIs for cytokines and chemokines by qPCR (**F**). Cell lysates from donor MH6352 cells only were assayed in duplicate for IL-1β, IL-6, IL-8 and CCL3, while ML7362 was used for CCL4. ** *p* < 0.01.

To determine if upregulated cytokine and chemokine gene expression was also present at the protein level, enzyme-linked immunosorbent (ELISA) analysis for IL-1β, IL-6, IL-8, CCL3 and CCL4 was performed at 24 h and MOIs = 1:100 and 1:1000 (Figure 6). 

The ELISA results at different MOIs mirrored those obtained with qPCR in regard to the demonstration of the upregulated expression of each inflammatory gene over mock infections (Figure 6). Furthermore, differences in gene expression levels between MOIs were also broadly reflected at the protein level; in particular, a drop in gene expression at MOI = 1:1000 vs. 1:100 for CCL3 was detected as reduced levels of CCL3 protein (Figure 6).

### 2.6. Effect of Clindamycin on Inflammatory Response 

**Figure 7:**
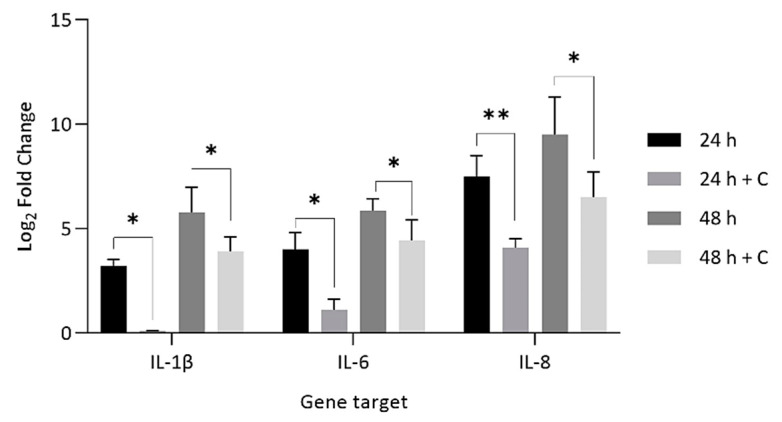
Comparison of log_2_ fold gene expression changes for IL-1β, IL-6 and IL-8 in NP-derived disc cells infected with *C. acnes* (MOI = 1:1000) versus cells pre-treated with clindamycin (+C) (0.25 µg/mL) for 60 min prior to *C. acnes* infection (MOI = 1:1000). * *p* < 0.05; ** *p* < 0.01.

To mimic a scenario of prophylactic antibiotic use before disc surgery and tissue excision, disc cells were pre-treated for 1 h with 0.25 µg/mL of clindamycin before inoculation with *C. acnes* at MOI = 1:1000 and measurement of IL-1β, IL-6 and IL-8 (Figure 7). 

Statistically significant reductions in gene expression for all three inflammatory molecules in samples where disc cells where pre-treated with antibiotics prior to infection were observed vs. non-antibiotic-treated infections (Figure 7). At 24 h and 48 h, fold changes decreased by 86–90% and 63–88%, respectively, after antibiotic pre-treatment. 

### 2.7. Neurotrophic Factor Gene Expression

**Figure 8:**
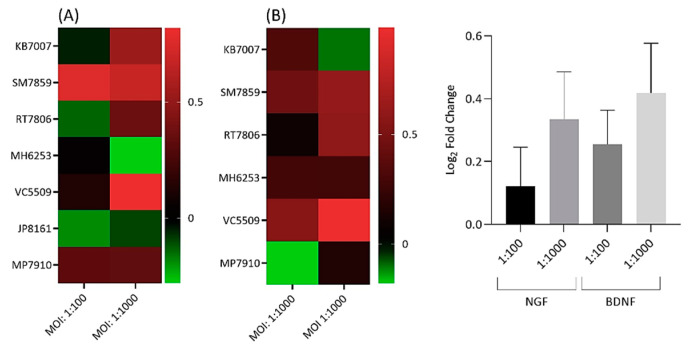
Heatmap and bar chart of log_2_ fold gene expression changes for nerve growth factor (NGF) (**A**) and brain-derived neurotrophic factor (BDNF) (**B**) in *C. acnes*-infected NP disc cells (MOIs = 1:100 and 1:1000). Heatmap values are individual changes (3 × biological replicates), while the bar chart represents average donor changes. NGF is based on seven cell donors and BDNF on six. Side bars represent the log_2_ fold-change scale.

For measurement of NGF and BDNF, the 48 h time point for MOIs = 1:100 and 1:1000 was utilised. Statistically significant, but moderate, levels of gene dysregulation for both neurotrophic factors were observed with infected cells over controls (Figure 8A,B).

As seen previously, results were patient specific (Figure 8A,B). Overall, higher expression changes were detected at MOI = 1:1000 versus 1:100, but this did not reach statistical significance (Figure 8). With NGF, the best response was observed with SM7859 cells, which gave consistent results at both MOIs, and KB7002 and VC5509 cells, which displayed significantly enhanced expression at MOI = 1:1000 only (Figure 8A,B). SM7859 cells, along with VC5509 cells, gave the best results for BDNF at both MOIs, while RT7806 cells demonstrated significantly increased expression at MOI = 1:1000 only (Figure 8A,B).

## 3. Discussion 

Overall, our qPCR and ELISA data clearly demonstrate the capacity of *C. acnes* to stimulate the expression of key cytokines, chemokines and growth factors directly from native NP-derived disc cells. In this regard, our work is consistent with a number of more recent studies which have now also revealed an inflammatory response of host disc cells to this bacterium, mediated in part via the Toll-like receptor (TLR) 2/4 pathway [38]; TLR activation has been linked to tissue degeneration and NGF regulation in IVDs [39,40,41]. We also see similar induction of inflammatory responses in keratinocytes by *C. acnes* activation of TLR signalling [42,43]. None of the small number of retrieved tissue samples analysed displayed any evidence of prior infection, indicating that the cells were likely *C. acnes* naive; the absence in 7/8 patients of type I Modic changes, which have been described as predictive of a *C. acnes*-positive culture [44], was also consistent with our microbiology results. Host responses to infection were variable and depended on the patient, gene target, MOI and time course. Previous investigations of disc cell response to *C. acnes* have utilised MOIs of 1:10 and 1:100 [15,38,45]. In our study, we also used an MOI of 1:100, but a higher MOI of 1:1000 was also investigated for its in vitro effects. As NP cells make up a small proportion of disc tissue mass, we acknowledge that it is very likely these MOIs are higher than levels found in patients. Nevertheless, our major objective at this point was to generate a clear dose response from our host cells, given the in vitro nature of our analysis and its inherent limitations, albeit offset somewhat by the use of primary cells. Similarly, other in vitro models of *C. acnes* infection, including those related to sarcoidosis, prostate and bone disease, have, for similar reasons, used MOIs which are likely to be much higher than those found in vivo, including MOIs up to 1:1000 [46,47,48,49].

An interesting observation from our studies was the presence of *C. acnes* within infected NP-derived disc cells, which, to our knowledge, has not been described before. At this point, it is unclear whether the intracellular *C. acnes* remained viable and could replicate or escape from the host cell or was simply the result of unprofessional phagocytic activity, as previously shown for human NP cells against *Staphylococcus aureus*, [50,51]. These will be important points to address moving forward, as intracellular survival in vivo, especially when combined with extracellular biofilm formation, would likely facilitate chronic persistence within the disc. The capacity of *C. acnes* to behave as an intracellular pathogen amongst a number of different cell types is well known, including human osteoblast cells [46,47,48,49,52]. The bacterium has also been identified intracellularly within sarcoid and prostate cancer tissue, where cell-wall-deficient or L-forms of the bacterium have been observed [53,54]. As a consequence, chronic intracellular *C. acnes* infection in the aetiology of disc degeneration is very plausible. 

Upon infection, we observed that disc cells did respond by upregulation of IL-1β production versus control samples, but the extent of expression varied between donors and in some cases required a higher MOI dose before significant increases were seen. Such personalised responses to infection are not unexpected when dealing with different primary cells, and this is consistent with recent previous reports [45]. Pre-sensitisation or trained immunity of disc cells has been suggested as a potential mechanism modulating responsiveness, with the presence of lumbar Modic changes (different types) at the same or adjacent level from where the cells were isolated being a key factor in whether they responded or not to infection [45]. In almost all our cases, donor samples did have Modic changes despite relative differences in their interactions with *C. acnes*. 

IL-1β is believed to play a central role in amplifying an inflammatory cascade within degenerate discs, including the expression of IL-6, IL-8, CCL3 and CCL4 [21,27]. As a result, and given their role in disc pathology, we also examined the expression levels of these inflammatory molecules in infected disc cells. Significant upregulation in expression over non-infected controls was detected and at levels which were greater overall than those observed with IL-1β; the responses were also donor specific. Under the in vitro conditions of our infection model, we noted a peak in the average inflammatory response for each target gene at either 24 or 48 h for each MOI. At the 24 h peaks, average expression levels at the next 48 h time point were found to drop off quite sharply in the cases of IL-6 and IL-8. Furthermore, we could only identify a trend or correlation between levels of IL-1β expression and those of IL-6, CCL3 and CCL4 in the very early infection phase (3 h) and at high bacterial loads (MOI = 1:1000). The upregulation of these pro-inflammatory molecules within the environment of the in vitro infection model therefore appeared to reflect other factors, including direct bacterial interactions and alternate cytokine stimuli. Nevertheless, future mechanistic studies with IL-1 receptor-blocking antibodies will enable us to better understand how IL-1β induction by *C. acnes* from disc cells could modulate other inflammatory mediators. Previous investigations have shown that expression levels of IL-6, IL-8, CCL3 and CCL4 increase in degenerate IVDs where they appear to play pivotal roles in disc tissue remodelling and nerve sensitisation [55,56,57]; furthermore, serum levels of IL-6, IL-8 and CCL3 are elevated in patients with disc degeneration, and the release of a cytokine cocktail from disc cells can also activate a pro-inflammatory response from bone marrow cells, which could potentially lead to the development of Modic changes [45,58,59]. 

The release of cytokines, especially IL-1β, can also induce the expression of NGF and BDNF in disc and immune cells, supporting nerve in-growth and increased pain sensitivity during degeneration [29,32]. Studies have found that NP cells isolated from degenerative, but not non-degenerative, discs stimulate both neurite length and the percentage of neurite-expressing cells via NGF and BDNF secretion, with BDNF appearing to play a more dominant role [60]. Upon infection of NP-derived cells, we were also able, for the first time, to demonstrate a significant upregulation in NGF and BDNF levels over controls, which indicates that, in vivo, *C. acnes* has the potential to stimulate neurotrophic factors which trigger neo-innervation. Furthermore, our results provide a possible mechanistic explanation for the observation of increased expression of genes related to peptidergic nerve fibres (substance P, calcitonin gene-related peptide) in rat lumbar IVD inoculation with *C. acnes* [61].

Previously, we reported that the positively charged antibiotic clindamycin performed better than negatively charged cefazolin and vancomycin at penetrating the intervertebral discs of patients undergoing spinal surgery [62]. Similar results have been described in rabbits, where the presence of infection was also shown to enhance clindamycin’s distribution within NP tissue [63]. The use of such antibiotics for spinal surgery prophylaxis is critical to prevent downstream infections which may arise from contamination of the surgical wounds with skin microbiota [64] and also to suppress any potential reservoirs of existing occult *C. acnes* infection in disc tissue, which could be disseminated during the surgical procedure. A one-off pre-treatment of our NP-derived cells with clindamycin prior to infection resulted in significant reductions in the inflammatory response versus non-antibiotic-treated cells, demonstrating, at least in vitro, the clinical efficacy against *C. acnes* in NP tissue. As clindamycin is primarily bacteriostatic, the results likely reflect a combination of inhibited bacterial growth over the time course examined, combined with modulating effects on cytokine induction. Although the effect of clindamycin on intracellular *C. acnes* was not investigated in our study, previous work has found the antibiotic can also suppress intracellular bacterial growth of various organisms [65]. With its recognised anti-bacterial effects against *C. acnes*, combined with its anti-inflammatory properties, good penetration of IVDs and capacity to inhibit a nociceptive response [66], clindamycin could, in principle, prove a good choice for the treatment of *C. acnes*-related DDD in CLBP, although it may be compromised somewhat by colonisation as a biofilm [10,67]. 

Previous studies have found that specific lineages of *C. acnes* display differences in their virulence properties, disease associations and interactions with various cell types [33,68]. While strains crossing a range of different *C. acnes* phylogroups have been isolated from surgically removed disc tissue, it still remains unclear whether there are degeneragenic strains with a heighted capacity to cause pathogenic remodelling of discs versus those whose presence is more benign or passive. In this study, we confined our host response studies to one strain isolated from a herniated disc, but it is probable that with other strains, we may see different inflammatory profiles, especially in relation to IL-1β, a pivotal and seemingly master cytokine in DDD. The ability of *C. acnes* to stimulate degenerative disc changes and low back pain is, therefore, likely to be multifaceted and dependent not only on its ability to colonise damaged discs but also on the nature of the strain(s) involved and the degree of the host response; in the latter case, this may reflect, in part, genetic susceptibility [5], as has been shown with acne [69,70].

## 4. Methods and Methods

### 4.1. Study Participants

Human IVD tissue samples containing degenerative disc cells were obtained from eight patients who underwent microdiscectomy at the University Hospital Brno (Brno, Czech Republic). These included four males and four females, with a mean age of (36–62 years) (Table 1). Disc degeneration was graded using the Pfirrmann scheme [71] (Table 1). Only one patient (KB7007) had a previous spinal procedure (epidural).

### 4.2. Isolation and Culture of Human, NP-Derived Primary Cells from Degenerative Discs

Fresh, surgically removed IVD tissue samples were collected under sterile conditions and processed immediately. The surgeons made every effort to ensure only NP tissue was provided, but any remaining identifiable organised fibre structures indicative of annulus fibrosus were removed in the laboratory and the tissue viewed microscopically to ensure it was comprised of mostly all NP cells (>90%) based on morphological and cellular features (data not shown). The tissue was then cut into small pieces using sterile, individually packaged scalpels and placed in sterile Petri dishes before digestion overnight at 37 °C with collagenase A (MilliporeSigma, Mannheim, Germany); some sections were retained for microbiological analysis. Cell suspensions were then filtered through a 40 µm cell strainer (MilliporeSigma) to remove any undigested tissue and obtain a uniform single-cell suspension before centrifugation at 1000 rpm for 5 min. To favour NP cell expansion over any potential macrophage or stromal cells, the resulting cell pellets were resuspended and washed in Dulbecco’s Modified Eagle Medium Nutrient Mixture F-12 (1 × 1:1 DMEM:F12) (Thermo Fisher Scientific, Waltham, MA, USA), supplemented with 10% (*v*/*v*) foetal bovine serum, penicillin (200 U/mL) and streptomycin (100 units/mL) (Thermo Fisher Scientific), before seeding tissue culture flasks. Cells were expanded at 37 °C in a humidified atmosphere containing 5% CO_2_ and maintained in monolayer culture, with 2–3 passages, prior to infection. Growth media was changed every 3–4 days after adhesion. 

### 4.3. Bacterial Culture from Disc Tissue

To determine whether any of the retrieved disc tissue had evidence of bacterial infection, additional sections were placed into a Stomacher^®^ Micro Bag (Seward Ltd., Worthing, UK) containing 4 mL of Viande-Levure medium and homogenised using a Stomacher^®^ 80 blender (Seward) under aseptic conditions (Class 2 safety hood). The resulting homogenates (100 μL aliquots) were then used to inoculate plates containing Wilkins–Chalgren anaerobic agar supplemented with 7% (*v*/*v*) sheep’s blood and vitamin K (HiMedia Laboratories, Mumbai, India) before incubation for 14 days at 37 °C in a Concept 400 anaerobic workstation (Ruskinn Technology, Bridgend, UK) under an atmosphere of 80% N_2_, 10% CO_2_ and 10% H_2_. Identical aliquots of the homogenate were also cultured aerobically for 7 days on Columbia blood agar (ThermoFisher Scientific, Waltham, MA, USA) at 37 °C for the detection of aerobic bacteria. 

### 4.4. Bacterial Strains for Infection Studies

For our infection studies, we used two *C. acnes* strains, *C. acnes* subsp. *acnes* (type IA_1_) and *C. acnes* subsp. *defendens* (type II), which were previously isolated under strict aseptic conditions from heavily infected herniated disc tissue [10]. Strains were cultured anaerobically, as described in Section 4.3.

### 4.5. Detection of Intracellular Bacteria

NP-derived disc cells (KB7007) were seeded in 6-well plates at a density of 10^5^ cells per well (antibiotic-free) and allowed to attach overnight. A fresh culture of *C. acnes* type IA_1_ was fluorescently labelled (in the dark) with 2 mL of 10 μM carboxyfluorescein succinimidyl ester (CFSE) in sterile phosphate buffered saline (PBS) for 1 h at 37 °C with shaking. The bacterial cells were then washed twice in sterile PBS before inoculation of disc cells at MOI = 1:100 and 1:1000 for 24 and 48 h. Images were acquired using a confocal Zeiss LSM 800 microscope with a Plan-Apochromat 40× NA 1.2 W objective (Carl Zeiss Microscopy GmbH, Oberkochen, Germany) and laser lines of 488 nm (CFSE) and 561 nm. The latter detected Calcein Red-Orange (ThermoFisher), which was used (at 1 μM) as a dye for NP cell viability (retained by live cells).

### 4.6. Infection of NP-Derived Disc Cells for Cytokine, Chemokine and Neurotrophic Factor Expression Analysis

As before, cells were seeded in 6-well plates at a density of 10^5^ cells per well (antibiotic-free) and allowed to attach overnight. Biological replicates (triplicates) were then infected with a freshly cultured *C. acnes* strain (type II) at an MOI = 1:100 and 1:1000. For the measurement of IL-1β, IL-6, IL-8 (CXCL-8), CCL3 and CCL4, time points of 3, 24 and 48 h were analysed. Treatment of cells with *Escherichia coli* lipopolysaccharide (LPS) (200 ng/µL; MillliporeSigma) served as an internal pro-inflammatory positive control for each experiment, while cells cultured in the absence of *C. acnes* acted as negative or mock controls for each time point. LPS-induced responses were measured at the 3 and 24 h time points only to demonstrate the pro-inflammatory response of the cells to a TLR agonist and to confirm our qPCR detection methods. For measurement of NGF and BDNF, the 48 h time point was used across the different primary cells. To investigate the effect of prior antibiotic treatment of cells on IL-1β, IL-6 and IL-8 inflammatory responses to infection, NP-derived cells were pre-treated for 1 h with 0.25 µg/mL of clindamycin before inoculation with *C. acnes* at an MOI = 1:1000 for 24 and 48 h. Infection in the absence of clindamycin served as a negative control. At the end of all time points, cells were washed and then harvested into Qiazol lysis reagent (Qiagen; Hilden, Germany) and radioimmunoprecipitation assay (RIPA) buffer (MilliporeSigma) for downstream RNA isolation. Cell culture supernatants were sterile-filtered and stored at −80 °C for ELISA.

### 4.7. RNA Isolation

RNA was extracted from mock-infected and infected cells using the Direct-zol RNA kit (Zymo Research, Irvine, CA, USA), as described in the manufacturer’s instructions. The concentration and purity of RNA were determined at 260 and 280 nm using a NanoDrop 2000 (Thermo Scientific).

### 4.8. Quantitative PCR (qPCR)

Total RNA was subjected to reverse transcription using the High-Capacity cDNA Reverse Transcription Kit (Applied Biosystems, Waltham, MA, USA). Reverse transcription was performed using the following temperature sequence: 10 min at 25 °C, 2 h at 37 °C and 5 min at 85 °C. qRT-PCR was performed using the TaqMan Gene Expression Master Mix (Applied Biosystems) and commercial primer/probe assays (ThermoFisher). These included IL-1β (Hs01555410_m1), IL-6 (Hs00174131_m1), IL-8 (CXCL-8) (Hs00174103_m1), CCL3 (HS00234142_m1), CCL4 (Hs99999148_m1), NGF (Hs00171458_m1) and BDNF (Hs02718934_s1). All experiments were performed using the real-time QuantStudio 12K Flex system (Thermo Fisher Scientific, Waltham, MA, USA), with glyceraldehyde phosphate dehydrogenase (GAPDH) as an internal housekeeping reference gene for normalisation; this gene has previously been used as an internal control for expression analysis of disc cells [38]. The thermal cycling program was as follows: 1 cycle of 94 °C for 10 min and 40 cycles consisting of 95 °C for 15 s and 60 °C for 60 s. Each sample was run in triplicate. The fold change in the normalised gene expression between mock and infected disc cells was calculated using the 2^−ΔΔCt^ method.

### 4.9. ELISA

To confirm qPCR expression data at the protein level, 24 h cell lysates in RIPA buffer (MilliporeSigma) were assayed, in duplicate, using commercially available ELISA kits (MilliporeSigma) for human IL-1β (RAB0273A), IL-6 (RAB0306), IL-8 (RABIL8A), CCL3 (RAB0073) and CCL4 (RAB0075). For the analysis of IL-1β, IL-6, IL-8 and CCL3, donor MH6253 cells were selected for analysis, while ML7362 cells were used for CCL4. These cell preparations were chosen based on their gene expression differences between MOIs for the various inflammatory molecules.

### 4.10. Statistical Analyses

Data were analysed using Student’s *t*-test (two-tailed), one-way ANOVA with Tukey’s post hoc multiple comparison and the Pearson correlation coefficient test (two-tailed). Data were presented as the mean ± standard error of the mean. Statistical significance was set at *p* ≤ 0.05. Statistical analysis was performed using GraphPad Prism 9.

## 5. Conclusions

This study further demonstrates the capacity of *C. acnes* to activate and stimulate an inflammatory response from native NP-derived disc cells, as well as an upregulation of neurotrophic growth factors, which, if occurring chronically in vivo, may be important in the context of Modic changes, neo-innervation and pain development. Such host responses are also likely to be further enhanced in vivo as extracellular matrix (ECM) breakdown products resulting from bacterial action, such as hyaluronic acid fragments, will also amplify the inflammatory cascade from disc cells [72]. While increases in IL-1β expression from disc cells upon in vitro infection were observed, levels varied between donors, thus indicating that the capacity of *C. acnes* infection to strongly induce IL-1β within degenerative discs depends on key host factors. However, in those patients where a heightened response to *C. acnes* does occur, we can easily envisage how disc infection could drive or enhance degeneration via increased levels of IL-1β production, alongside other inflammatory mediators and host-interacting molecules, from IVD cells.

In addition to continued mechanistic-based studies to explain how *C. acnes* could stimulate disc degeneration, one area which requires attention moving forward is the development of novel and ideally non-invasive methods to identify CLBP patients with *C. acnes-*infected/*C. acnes-*colonised discs. Such diagnostic tools would help to improve the effect size of clinical trials for the evaluation of drug treatments on the eradication of the bacterium and its association with symptom alleviation, thus providing further evidence for or against a role in the aetiology of DDD.

## Figures and Tables

**Table 1:** Characteristics of patients undergoing spinal surgery.

Primary Cell Designation	Gender	Age	Herniation *	Disc Segment	Oswestry Core	Modic Changes	Pfirrman Grade
KB7007	M	47	P	L5/S1	42	II	III
SM7859	F	39	S	L4/5	56	N/A	N/A
RT7806	M	39	E	L4/5	34	II	IV
MH6253	F	55	E	L4/5	44	I+II	III
VC5509	M	62	S	L4/5	28	II	IV
JP8161	F	36	S	L5/S1	66	II	IV
ML7362	F	44	E	L4/5; L5/S1	32	II; II	V;V
MP7910	M	38	S	L5/S1	34	II	IV

* P, pronation; E, extrusion; S, sequestration.
